# Lithium augmentation of ketamine increases insulin signaling and antidepressant-like active stress coping in a rodent model of treatment-resistant depression

**DOI:** 10.1038/s41398-021-01716-w

**Published:** 2021-11-25

**Authors:** J. Blair Price, Clarissa G. Yates, Brooke A. Morath, Sam K. Van De Wakker, Nathanael J. Yates, Kim Butters, Mark A. Frye, Sean L. McGee, Susannah J. Tye

**Affiliations:** 1 Department of Psychiatry and Psychology, Mayo Clinic Depression Center, Mayo Clinic, 200 1st St SW, Rochester, MN 55905 United States; 2 Institute for Mental and Physical Health and Clinical Translation (IMPACT), Metabolic Research Unit, School of Medicine, Deakin University, 75 Pigdons Road, Waurn Ponds, VIC 3216 Australia; 3 Department of Neurologic Surgery, Mayo Clinic, 200 1st St SW, Rochester, MN 55905 United States; 4 Queensland Brain Institute, The University of Queensland, St Lucia, QLD 4072 Australia; 5 Department of Psychiatry, Emory University, Atlanta, GA 30322 United States; 6 Department of Psychiatry, University of Minnesota, Minneapolis, MN 55455 United States

**Keywords:** Molecular neuroscience, Neuroscience, Predictive markers

## Abstract

Lithium, a mood stabilizer and common adjunctive treatment for refractory depression, shares overlapping mechanisms of action with ketamine and enhances the duration of ketamine’s antidepressant actions in rodent models at sub-therapeutic doses. Yet, in a recent clinical trial, lithium co-treatment with ketamine failed to improve antidepressant outcomes in subjects previously shown to respond to ketamine alone. The potential for lithium augmentation to improve antidepressant outcomes in ketamine nonresponders, however, has not been explored. The current study examined the behavioral, molecular and metabolic actions of lithium and ketamine co-treatment in a rodent model of antidepressant resistance. Male Wistar rats were administered adrenocorticotropic hormone (ACTH; 100 µg/day, i.p. over 14 days) and subsequently treated with ketamine (10 mg/kg; 2 days; *n* = 12), lithium (37 mg/kg; 2 days; *n* = 12), ketamine + lithium (10 mg/kg + 37 mg/kg; 2 days; *n* = 12), or vehicle saline (0.9%; *n* = 12). Rats were subjected to open field (6 min) and forced swim tests (6 min). Peripheral blood and brain prefrontal cortical (PFC) tissue was collected one hour following stress exposure. Western blotting was used to determine the effects of treatment on extracellular signal-regulated kinase (ERK); mammalian target of rapamycin (mTOR), phospho kinase B (Akt), and glycogen synthase kinase-3ß (GSK3ß) protein levels in the infralimbic (IL) and prelimbic (PL) subregions of the PFC. Prefrontal oxygen consumption rate (OCR) and extracellular acidification rates (ECAR) were also determined in anterior PFC tissue at rest and following stimulation with brain-derived neurotrophic factor (BDNF) and tumor necrosis factor α (TNFα). Blood plasma levels of mTOR and insulin were determined using enzyme-linked immunosorbent assays (ELISAs). Overall, rats receiving ketamine+lithium displayed a robust antidepressant response to the combined treatment as demonstrated through significant reductions in immobility time (*p* < 0.05) and latency to immobility (*p* < 0.01). These animals also had higher expression of plasma mTOR (*p* < 0.01) and insulin (*p* < 0.001). Tissue bioenergetics analyses revealed that combined ketamine+lithium treatment did not significantly alter the respiratory response to BDNF or TNFα. Animals receiving both ketamine and lithium had significantly higher phosphorylation (*p*)-to-total expression ratios of mTOR (*p* < 0.001) and Akt (*p* < 0.01), and lower ERK in the IL compared to control animals. In contrast, *p*mTOR/mTOR levels were reduced in the PL of ketamine+lithium treated animals, while *p*ERK/ERK expression levels were elevated. Taken together, these data demonstrate that lithium augmentation of ketamine in antidepressant nonresponsive animals improves antidepressant-like behavioral responses under stress, together with peripheral insulin efflux and region-specific PFC insulin signaling.

## Introduction

Major depressive disorder (MDD) affects over 300 million people and is estimated to be the leading cause of disease burden globally by 2030 [1]. Broadly speaking, antidepressants are effective in treating this illness. Yet, approximately one-third of patients fail to receive therapeutic benefit after four sequential trials of antidepressant therapy and are considered to have a more persistent form of MDD, often defined as treatment-resistant depression (TRD) [2]. The emergence of novel, rapid-acting antidepressants such as ketamine over the last decade has provided valuable new treatment opportunities for individuals with TRD, together with unique insight into the mechanisms governing antidepressant response at the cellular and molecular level. Such treatments have underscored the key role that neurotrophic signaling and synaptic plasticity play at the cellular level to enable antidepressant behavioral efficacy [3–5]. Ketamine and esketamine elicit these rapid effects via direct stimulation of glutamatergic neurotransmission to promote brain derived neurotrophic factor (BDNF) release, neurotrophic signaling, and consequent dendritic spine growth [6–9]. Esketamine’s FDA approval marks the first approval in decades of an antidepressant featuring a novel mechanism that is known to address treatment-resistant forms of depression [6, 9]. However, despite the excitement that has accompanied the introduction of these rapid antidepressants into psychiatric practice, a significant portion of individuals with TRD remain underserved and do not receive clinical benefit, with clinical trials variably citing cumulative response rates between 29–90% [10]. This raises the question as to whether key physiological mechanisms might functionally block the adaptive upregulation of neurotrophic signaling and, if so, can these molecular targets be otherwise engaged with adjunctive treatment strategies? Elucidating these mechanism will enable us to more precisely align complementary treatments to facilitate neurotrophic and behavioral responses to ketamine.

Critical to ketamine’s mechanism of action is the rapid upregulation of α-amino-3-hydroxy-5-methyl-4-isoxazolepropionic acid (AMPA) receptors and BDNF to promote neurotrophic signaling via activation of mammalian target of rapamycin (mTOR) and inhibition of glycogen synthase kinase-3 ß (GSK3ß). These molecular cascades, in turn, have been shown to functionally promote the development of new synaptic connections to enhance connectivity between mood-related brain regions [4, 11, 12]. Lithium also activates this molecular pathway, which in addition to BDNF, is stimulated by other endogenous growth factors, including insulin [3]. Consequently, lithium is a promising candidate for adjunctive treatment to ketamine and has shown potential as an augmentative agent for other antidepressant approaches for TRD [13, 14]. In a rodent model of TRD, lithium augmentation of imipramine was shown to promote central and peripheral insulin signaling, which was directly correlated with antidepressant behavioral response [15]. Lithium, a monovalent cation is well-established to activate neuroprotective and neurotrophic cellular cascades [16–19], including the direct modulation of bioenergetic factors (i.e., increased insulin-like growth factor (IGF) expression [20, 21], protection of mitochondrial health, cellular energy metabolism and antioxidant defense [22, 23], and stimulation of neurotrophic molecular cascades (mTOR and GSK3ß) [16, 17, 24, 25]). Collectively, these actions work together to promote cell growth and antiapoptotic effects [16]. Downstream of these mechanisms, both lithium and ketamine initiate neurotrophic cascades via activation of MAPK kinase (MEK)/extracellular-receptor kinase (ERK) and cAMP response element-binding protein (CREB), which provide bioenergetic stimuli for neural and synaptic plasticity, and play a critical role in enabling antidepressant response to ketamine [16, 26, 27].

Given the overlapping physiological impacts of these treatments, we have examined whether adjunctive lithium would enhance antidepressant response to ketamine, in a subset of ketamine nonresponsive rodents. Although the interactive effects of ketamine and lithium have been examined in prior research [28, 29], this study is the first to investigate ketamine and lithium’s additive antidepressant-like effects in a rat model of ketamine treatment resistance. We further characterize bioenergetic and neurotrophic biomarkers associated with antidepressant response outcomes.

## Methods

Male Wistar rats (*n* = 48) weighing 150–250 g were used in this study. Animals were housed in pairs in cages within a room with controlled temperature (20–22 °C) on a 12 h light-dark cycle (lights: on 07:00; off 19:00). Food and water were available *ad libitum*. Animals began the experimental protocol at five weeks of age following a three-day acclimatization period. All procedures were approved by the Mayo Clinic Institutional Animal Care and Use Committee and performed according to the National Institutes of Health guidelines.

### Drugs

Drug treatments used in this study included adrenocorticotropic hormone ACTH-[1–14, 16–21, 24, 30–32] (100 ug/day; 1 ml), ketamine (10 mg/kg; 0.5–0.7 ml), lithium (37 mg/kg; 0.15–0.25 ml), control vehicle 0.9% saline, and FatalPlus^®^ (0.70 ml; constituents: pentobarbital sodium 390 mg/ml; propylene glycol 0.01 mg/ml; ethyl alcohol 0.29 mg/ml; benzyl alcohol (preservative) 0.20 mg/ml). All drugs were delivered via intraperitoneal (i.p.) injection.

### Experimental procedure

Male Wistar rats were administered ACTH for 14 days, establishing an antidepressant-resistant phenotype as previously described [15, 24, 25]. On days 14 and 15, rats were administered ketamine (*n* = 12), lithium (*n* = 12), ketamine + lithium (*n* = 12), or control vehicle saline (*n* = 12). Treatment groups were assigned randomly by the experimenter. Rats were returned to home cages for 1-hour prior to the open field (day 14 only) and forced swim (day 15) tests to prevent acute psychomimetic effects of drug administration interfering with behavioral testing. Thirty minutes after behavioral testing, rats were euthanized via anesthetic overdose, and cardiac blood and brain tissue were collected. PFC tissue was excised from the brain and placed in a buffer to cryopreserve mitochondria (mannitol, 200 mM; sucrose, 50 mM; KH2PO4, 5 mM; EGTA, 1 mM; MOPS, 5 mM; BSA, 0.10%). The buffer was removed, and tissue was placed in a freezing buffer (mannitol, 200 mM; sucrose, 50 mM; KH2PO4, 5 mM; EGTA, 1 mM; MOPS, 5 mM; BSA, 0.10%; DMSO, 20%) and frozen at −80°. Remaining brain tissue and blood samples were frozen on dry ice and stored at −80 °C until later use.

### Behavioral testing

#### Open field test

The open-field test was conducted on day 14, 60 minutes after drug treatment. This test was conducted to examine locomotor behaviors in response to treatment, serving as a control for behavioral activity in the forced swim test on day 15. Animals were placed in the center of an open field arena (60 cm length × 60 cm width × 60 cm height) and allowed to move unimpeded for 6 minutes. Behaviors during this time were recorded by a video camera for later analysis using CleverSys Top Scan software. Behaviors of interest included *time spent in center of arena* and *total distance traveled*. Following open field testing, rats were returned to home cages before forced swim test training.

#### Forced swim test

The forced swim test is a tool with robust predictive validity for screening antidepressants and is designed to measure coping-like behaviors of animals following exposure to a novel stressful environment. The forced swim apparatus consisted of transparent plexiglass cylindrical tanks (45 cm height × 20 cm diameter) filled with water (23 °C) to a depth of 30 cm. On day 14, two hours after completion of the open field test, rats were exposed to a 15-min training session in which they were immersed in the apparatus. The following day, a 6-min test session was conducted for behavioral analysis. Sessions were recorded and footage was de-identified and analyzed by hand in 1-second intervals. Behaviors of interest included *immobility* and *time to immobility* (passive) behavior, together with *swimming* and *climbing* (active) behavior. The final 4 min of the test was used for analysis.

### Peripheral blood levels of insulin and mTOR

Commercially available enzyme-linked immunosorbent assay (ELISA) kits were used to determine insulin (ALPCO, 80-INSRT-E01, E10) and mTOR (LifeSpan BioSciences, Inc., LS-F32183) concentrations present in the plasma samples. All procedures were carried out per manufacturer instructions and guidelines.

### Prefrontal bioenergetics analyses

PFC tissue bioenergetics were analyzed using a Seahorse XF24–3 Bioanalyzer (Agilent) as we have previously described [33], with some minor modifications. PFC samples were biopsied with a 1 mm biopsy punch and tissues were placed into a Seahorse Islet Capture Plate, with mesh screens placed on top, in unbuffered DMEM containing 2 mM glucose as the sole substrate. The plate was placed in a non-CO_2_ incubator at 37 °C for 10 min prior to assay, which used 2-min mix, 2-min wait, and 2-min measurement cycles throughout the procedure. Two basal measurements were made before the injection of glucose, succinate, malate (final concentrations of 5 mM each), glutamate (final concentration 1 mM) and adenosine diphosphate (ADP; final concentration 2.5 mM). Another five measurements were obtained prior to the addition of either BDNF (Cloud Clone Corp; final concentration 50 ng/mL) or TNFα (Peprotech; final concentration 100 ng/mL), followed by another five measurements. The mean OCR and ECAR values across five measurements following substrate and ADP administration were expressed as a percentage of basal values. Similarly, the mean OCR and ECAR values across the five measurements following BDNF or TNFα were expressed as a percentage of substrate and ADP-stimulated values.

### Prefrontal protein signaling

#### Tissue preparation

A 1 mm biopsy punch was made from IL and PL regions of the medial PFC and placed into 1.5 ml Eppendorf tubes with100µl cold radioimmunoprecipitation assay (RIPA) buffer. Tissue lysis was performed by sonication for 10 seconds followed by centrifugation (1600 rpm, 20 minutes at 4 °C). The protein content of supernatant samples was estimated by protein assay (Pierce™ Coomassie Plus (Bradford) Assay Kit, Thermofisher Scientific) according to the manufacturer’s instructions. Samples were subsequently diluted to equal concentrations in RIPA buffer and then added 4:1 in sample buffer (Laemmli buffer supplemented with 10% β-mercaptoethanol) and heated at 95 °C for 10 min to denature proteins. Samples were loaded onto precast 4–12% Mini-PROTEAN^®^ TGX™ Precast Gels (Bio-Rad), with 1x Tris/Glycine/SDS running buffer (Bio-Rad) and electrophoresed at 200 V for 35 minutes. Protein Precision Plus Dual Color (Bio-Rad) provided molecular mass standards. Proteins were transferred onto nitrocellulose membranes using the Trans-Blot Turbo Transfer System (Bio-Rad).

#### Protein detection and densitometry analysis

Nitrocellulose membranes containing the transferred proteins were blocked in Intercept Blocking Buffer (LI-COR Bioscience) for 1-hour at room temperature. Membranes were probed with primary antibodies, including mouse anti-mTOR (1:1000; Cat# 4517), mouse anti-Akt (1:1000; Cat# 2920), mouse anti-GSK3β (1:1000; Cat# 9832), mouse anti-ERK (1:1000; Cat# 9107), rabbit anti-phospho mTOR (1:1000; Cat# 5536 S), rabbit anti-phospho Akt (1:1000; Cat# 4060 S), rabbit anti-phospho GSK3β (1:1000; Cat# 5558), rabbit anti-phospho ERK (1:1000; Cat# 4376) (all antibodies were purchased from Cell Signaling Technologies) diluted in Intercept Blocking Buffer + 0.2% Tween-20 and incubated overnight at 4 °C, before being exposed to secondary antibody (IRDye^®^ 680RD Donkey anti-Mouse IgG and IRDye^®^ 800CW Donkey anti-Rabbit IgG) (LI-COR Biosciences) for 1-hour at room temperature in the dark.

Membranes were scanned on the LI-COR Bioscience Odyssey CLx imaging system and imaged using LI-COR Image Studio software version 2.1.10. All densitometry analyses were performed using Image Studio Light version 5. The region of interest encircling each band was defined automatically. All bands at the correct molecular weight were analyzed as the signal for that target protein. Values for each protein were normalized to loading control β-tubulin (Abcam).

### Statistical analysis

Separate two-way analyses of variance (ANOVA) with subsequent Tukey’s honest significant difference (HSD) tests were used to analyze both behavioral and biochemical data. All calculations were performed using GraphPad Prism 8. The sample size (*n* = 12 per group) was chosen to provide 80% power to detect a group mean difference of 1 standard deviation across the 4 treatment conditions for each behavioral measure. Investigators conducting behavioral tests and tissue assays were blinded to animal treatment groups during testing, scoring and quantification procedures. Two blinded investigators hand-scored forced swim data and to ensure consistency.

## Results

### Behavioral data

#### Open field test

We examined the effects of treatment on behavior in the open field test, which quantifies locomotor behavior in an open arena. Behaviors are quantified as *total distance traveled* and *time spent in center of arena*. No significant group main effects, nor treatment interactions were observed, indicating treatments had no effect on locomotor activity.

#### Forced swim test

**Fig. 1:**
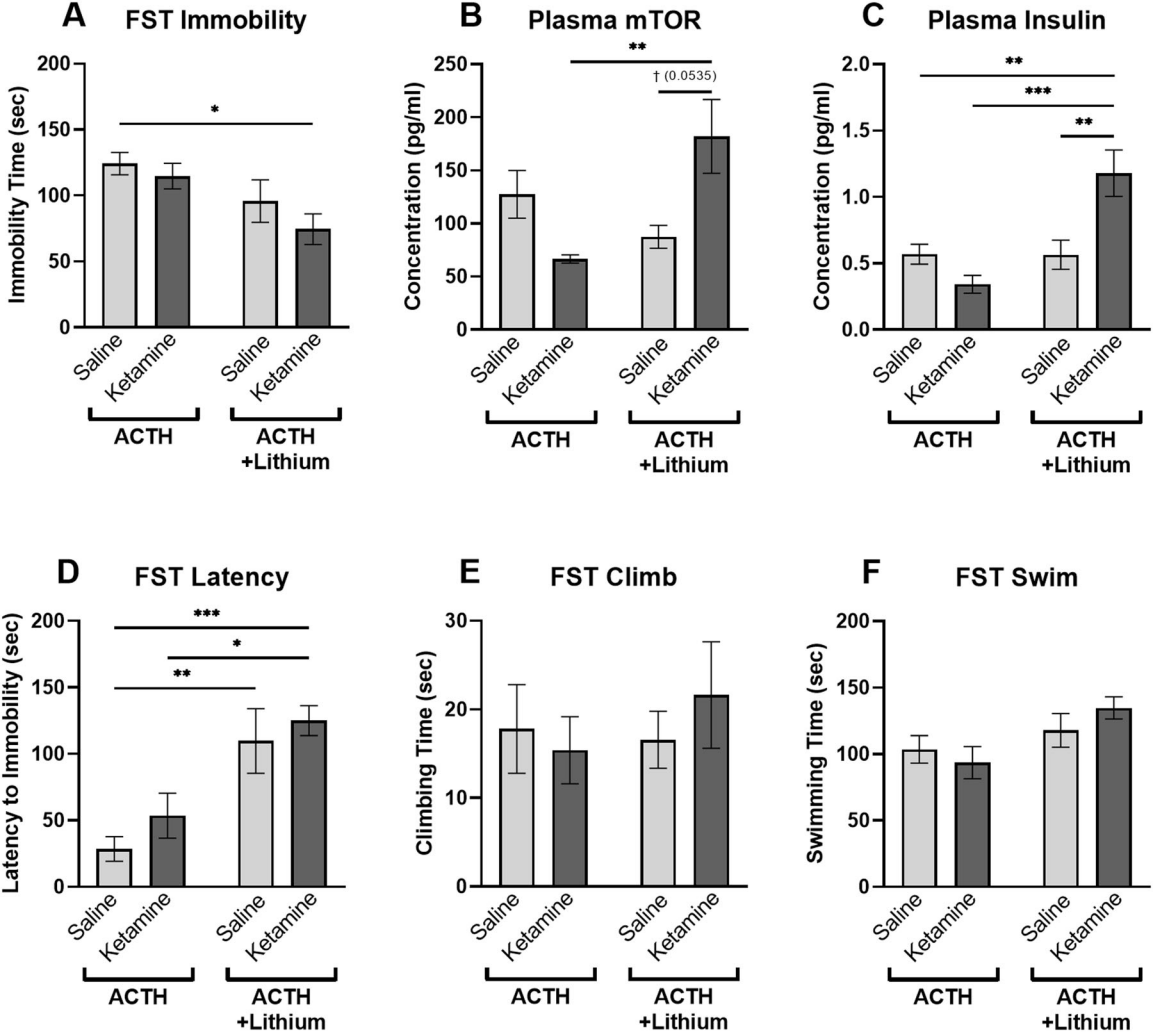
Behavioral and biochemical measures following treatment administration. Following administration of ACTH, ACTH + ketamine, ACTH + lithium, or ACTH + ketamine+lithium, antidepressant-like efficacy was determined according to time spent immobile (**A**), latency to immobility (**B**), swimming (**C**), or climbing (**D**) in the FST. In addition, biomarkers related to antidepressant effect were measured via ELISA, investigating expression of mTOR (**E**), GRIA1 (**F**), and insulin (**G**). ACTH adrenocorticotropic hormone, FST forced swim test, mTOR mammalian target of rapamycin, ELISA enzyme-linked immunosorbent assay, GRIA1 glutamate receptor, ^†^*p* < 0.06; *p < 0.05; **p < 0.01; ***p < 0.001.

A significant main effect of lithium treatment on immobility behaviors was found in the forced swim test (Fig. 1A; *p* = 0.0057; *F* = 8.878). Post hoc analyses revealed that animals treated with ACTH + ketamine+lithium displayed significantly less immobility than ACTH-controls (*p* = 0.0188). No significant differences were found when comparing ACTH-control animals to animals receiving ACTH + ketamine or ACTH + lithium. Additionally, a significant main effect of lithium treatment on latency to immobility was observed (Fig. 1D; *p* < 0.0001; *F* = 22.52). Post hoc multiple comparisons revealed that animals treated with ACTH + ketamine+lithium had significantly longer latency to immobility than ACTH-controls (*p* = 0.0010) and ACTH + ketamine (*p* = 0.0180) animals. Additionally, animals treated with ACTH + lithium had longer latency to immobility than ACTH-controls (*p* = 0.0060). No significant differences were found for climbing behaviors (Fig. 1E), while a significant main effect of lithium treatment on swimming behaviors was observed (Fig. 1F; *p* = 0.0189; *F* = 6.115). No treatment group differences were significant following post hoc multiple comparisons.

### Treatment effects on central and peripheral bioenergetic and molecular markers

#### Peripheral blood

##### Mammalian target of rapamycin

Plasma mTOR concentrations were measured to determine whether detectible changes in peripheral protein levels were observed between groups. When comparing all treatment groups together, a significant interaction effect of treatment was revealed (Fig. 1B; *p* = 0.0034; *F* = 10.09). Post hoc multiple comparisons were performed, finding significantly elevated expression of mTOR in rats treated with both ketamine and lithium compared to rats treated only with ketamine (*p* = 0.0096).

##### Insulin

Insulin levels in peripheral blood samples were elevated in rats treated with both ketamine and lithium compared to all other groups. A significant interaction effect (*p* = 0.0028; *F* = 10.27) and effect of lithium treatment (*p* = 0.0031; *F* = 10.05) was revealed when comparing insulin expression between all treatment groups (Fig. 1C). Post hoc multiple comparisons revealed significantly increased concentrations of insulin in animals receiving both ketamine and lithium compared to those receiving only ketamine (*p* = 0.0004), lithium (*p* = 0.0052), or vehicle (*p* = 0.0092) treatments.

#### Prefrontal bioenergetics

##### OCR and ECAR in response to substrates and ADP

**Fig. 2:**
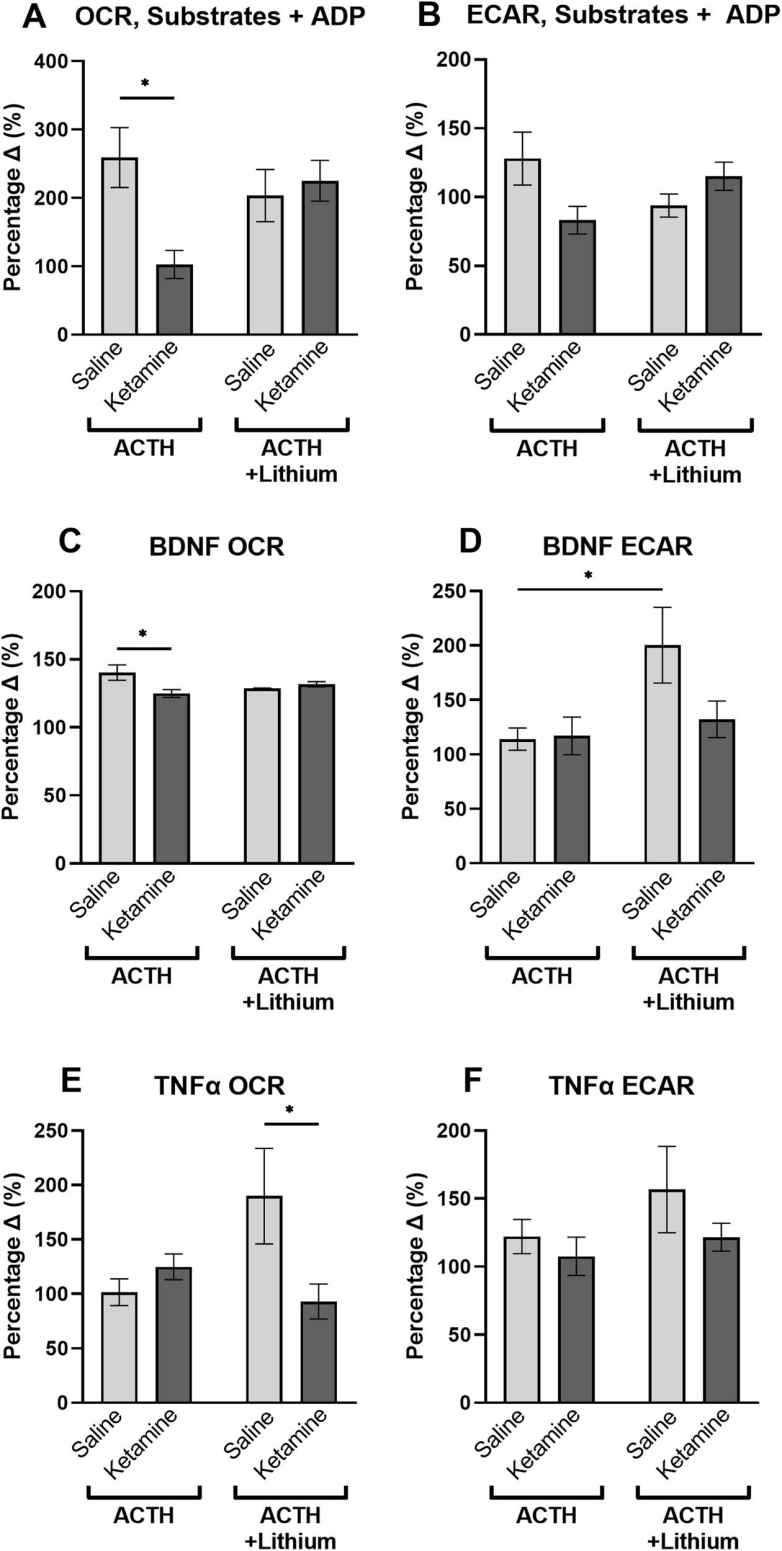
Mitochondrial OCR and ECAR responses to stimulation. Mitochondrial OCR and ECAR responses were determined following initial exposure to substrate and ADP challenge (**A**, **B**) and following exposure to biomarkers (BDNF, (**C**, **F**); TNFα (**D**, **G**); Wnt3a (**E**, **H**)). OCR oxygen consumption rate, ECAR extracellular acidification rate, ACTH adrenocorticotropic hormone, BDNF brain-derived neurotrophic factor, Wnt3a WNT family member 3a, TNFα tumor necrosis factor α, ADP adenosine diphosphate. *p < 0.05.

The addition of glucose, mitochondrial substrates, and ADP stimulates both oxidative and non-oxidative substrate utilization and is a measure of the bioenergetic capacity of a tissue. A significant interaction effect of treatment on OCR response was observed across all treatment groups (*p* = 0.0157; *F* = 6.103). Post hoc multiple comparisons found that relative to controls, the OCR response to substrates and ADP was lower in animals treated with ketamine (Fig. 2A; *p* = 0.0248). Similarly, a significant interaction effect of treatment on ECAR response was observed (*p* = 0.0145; *F* = 6.251). However, multiple comparisons revealed no differences between groups (Fig. 2B).

##### OCR and ECAR in response to BDNF

A significant interaction effect of BDNF stimulation on OCR response was observed (*p* = 0.0132; *F* = 7.000). Post hoc multiple comparisons revealed that ACTH + ketamine-treated animals had significantly reduced OCR compared to ACTH-controls (Fig. 2C; *p* = 0.0270). Further, a significant effect of lithium treatment on ECAR response to BDNF stimulation was observed (*p* = 0.0222; *F* = 5.981). Multiple comparisons revealed that ACTH + lithium-treated animals have significantly greater ECAR response compared to ACTH-controls (Fig. 2D; *p* = 0.0304).

##### OCR and ECAR in response to TNFα

Following TNFα stimulation, a significant interaction effect was observed on OCR response (*p* = 0.0168; *F* = 6.435). Among these groups, ACTH + ketamine+lithium-treated animals had significantly reduced OCR response relative to ACTH + lithium-treated animals (Fig. 2E; *p* = 0.0223). No significant differences were found when examining ECAR in response to TNFα stimulation (Fig. 2F).

#### Infralimbic prefrontal insulin signaling

##### Total and phosphorylated ERK1/2

**Fig. 3:**
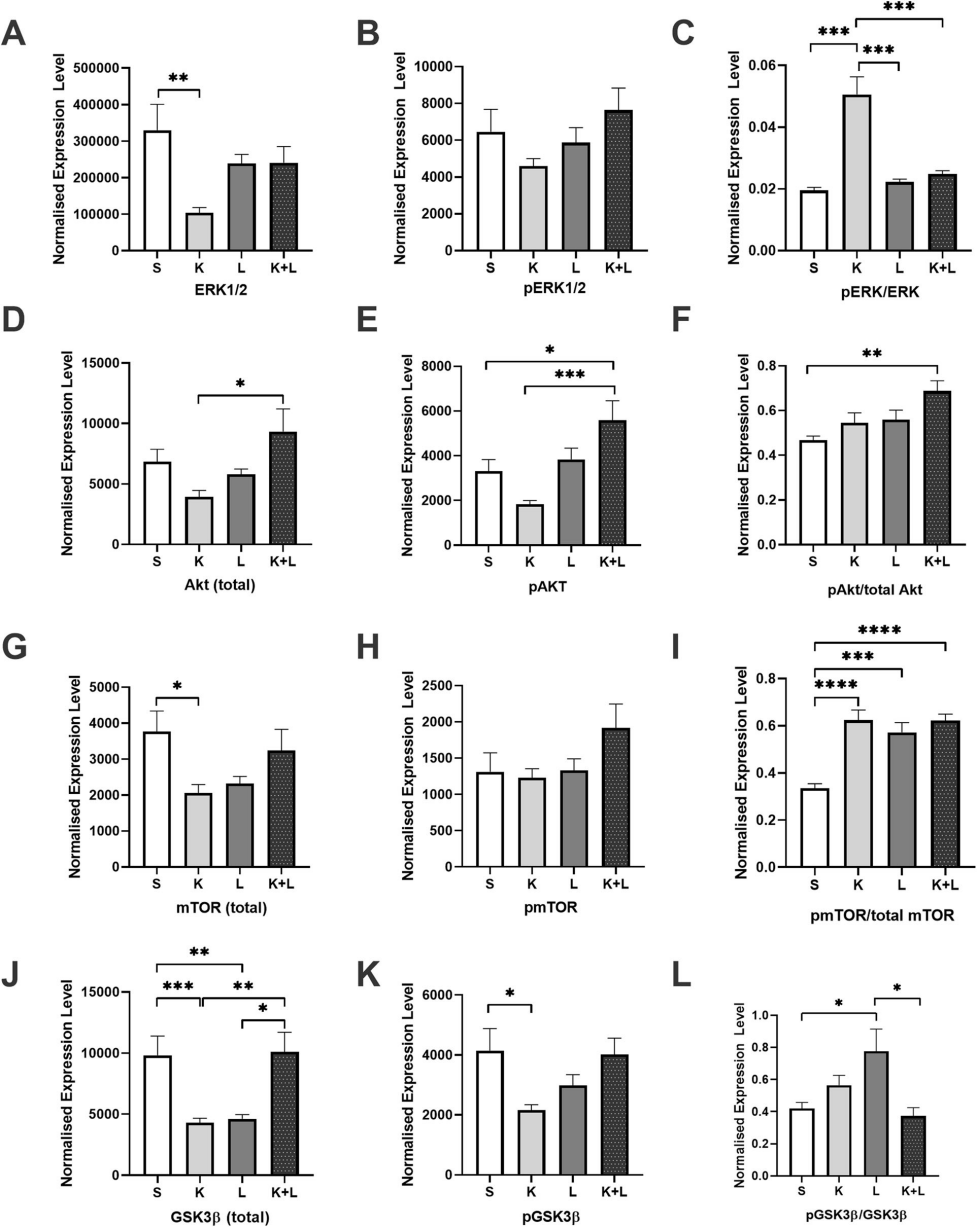
Infralimbic cortex protein expression in response to treatment. The effect of ketamine and lithium treatment on protein signaling profiles in the infralimbic cortex were examined via western blotting. Proteins of interest include total ERK1/2 (**A**), *p*ERK1/2 (**B**), *p*ERK1/2/ERK1/2 (**C**), total Akt (**D**), *p*Akt (**E**), *p*Akt/Akt (**F**), mTOR (**G**), *p* mTOR (**H**), and *p* mTOR/mTOR (**I**), GSK3ß (**J**), *p*GSK3ß (**K**), and *p*GSK3ß/GSK3ß (**L**). ERK1/2 extracellular signal-regulated kinases 1/2, *p* phosphorylated, Akt protein kinase B, mTOR mammalian target of rapamycin, GSK3ß glycogen synthase kinase-3 ß. Treatment groups: S: saline; K: ketamine; L: lithium; K + L: ketamine + lithium. *p < 0.05; **p < 0.01; ***p < 0.001; ****p < 0.0001.

A significant main effect of ketamine treatment (*p* = 0.0151; *F* = 6.402) was observed for ERK1/2 signaling in the IL PFC. A significant interaction effect (*p* = 0.0136; *F* = 6.628) was also observed. Post hoc multiple comparisons revealed that ACTH-controls had significantly less expression of total ERK1/2 compared to ACTH control animals administered saline (*p* = 0.0042) (Fig. 3A). No significant interaction effect of treatment was observed for *p*ERK1/2 (*p* = 0.0685; *F* = 7.105) (Fig. 3B). When examining the ratio of *p*ERK1/2 to total ERK1/2 (expressed as *p*ERK1/2/ ERK1/2), a significant effect of lithium (*p* < 0.005; *F* = 11.88) and ketamine (*p* < 0.0001; *F* = 25.30) treatment were observed, together with a significant interaction effect (*p* < 0.0005; *F* = 18.09). Post hoc multiple comparisons revealed that ACTH pretreated animals receiving ketamine expressed significantly higher *p*ERK1/2/ERK1/2 compared to ACTH-controls (*p* < 0.0001), ACTH + lithium (*p* < 0.0001), and ACTH + ketamine + lithium (*p* = < 0.0001) groups (Fig. 3C).

##### Total and phosphorylated Akt

When examining total Akt, a significant treatment interaction effect observed (*p* = 0.0087; *F* = 7.582), however lithium main effect was not significant (*p* = 0.0714; *F* = 3.42). Post hoc analyses revealed levels of Akt were significantly higher in ketamine and lithium co-treated animals relative to those only treated with ketamine (*p* < 0.05) (Fig. 3D). When examining *p*Akt expression, a significant main effect was observed for ketamine (*p* = 0.0006; *F* = 13.64), together with a significant treatment interaction (*p* = 0.0077; *F* = 7.815). Post hoc tests demonstrated the animals receiving both ketamine and lithium exhibited significantly higher levels of *p*Akt relative to control saline (*p* = 0.0358) and ketamine (*p* = 0.0003) treated groups (Fig. 3E). When examining the ratio of *p*Akt to Akt, a main effect of lithium (*p* = 0.006; *F* = 8.374) and ketamine (*p* = 0.0154; *F* = 6.373) treatment was observed. Post hoc multiple comparisons revealed that ACTH pretreated animals receiving both ketamine and lithium had significantly greater *p*Akt/Akt compared to those animals receiving only vehicle (*p* = 0.0025) (Fig. 3F).

##### Total and phosphorylated mTOR

A significant interaction effect was observed for mTOR across treatments (*p* = 0.0019; *F* = 10.94). Post hoc multiple comparisons revealed that ACTH-controls had significantly greater expression levels of mTOR compared to animals administered ketamine (*p* = 0.0186) (Fig. 3G). No significant main effects were observed for *p*mTOR (Fig. 3H). In contrast, a main effect for ketamine (*p* < 0.0001; *F* = 27.55) and lithium (*p* = 0.0017; *F* = 11:31) was observed for *p*mTOR/mTOR levels, together with a significant interaction effect (*p* = 0.0015; *F* = 11:50). Multiple comparisons revealed that relative to control animals the ratio of *p*mTOR/mTOR, indicative of mTOR activation, was significantly elevated in animals treated with ketamine (*p* < 0.0001), lithium (*p* = 0.0002), or the combination of ketamine with lithium (*p* < 0.0001) (Fig. 3I).

##### Total and phosphorylated GSK3ß

When examining GSK3ß, a main interaction effect of treatment was observed (*p* < 0.0001; *F* = 27.16). Post hoc multiple comparisons revealed that ACTH-pretreated control animals had significantly higher GSK3ß compared to animals treated with ketamine (*p* = 0.001) and lithium (*p* = 0.003). Animals treated with the combination (ketamine + lithium) also exhibited significantly elevated levels of GSK3ß compared to animals treated only with ketamine (*p* = 0.0041) or lithium (*p* = 0.011) (Fig. 3J). An interaction main effect was also present for *p*GSK3ß (*p* = 0.0041; *F* = 9.149), with post hoc analyses revealing expression was significantly reduced in ketamine treated animals relative to controls (*p* = 0.0359), with a trend apparent relative to those animals co-treated with ketamine and lithium (*p* = 0.0543) (Fig. 3K). Similarly, an interaction main effect was present for *p*GSK3ß/GSK3ß (*p* = 0.0016; *F* = 11.52), with expression levels significantly increased in lithium treated animals relative to controls (*p* = 0.0133), and animals receiving the combination of ketamine and lithium (*p* = 0.011) (Fig. 3L).

#### Prelimbic prefrontal insulin signaling

##### Total and phosphorylated ERK1/2

**Fig. 4:**
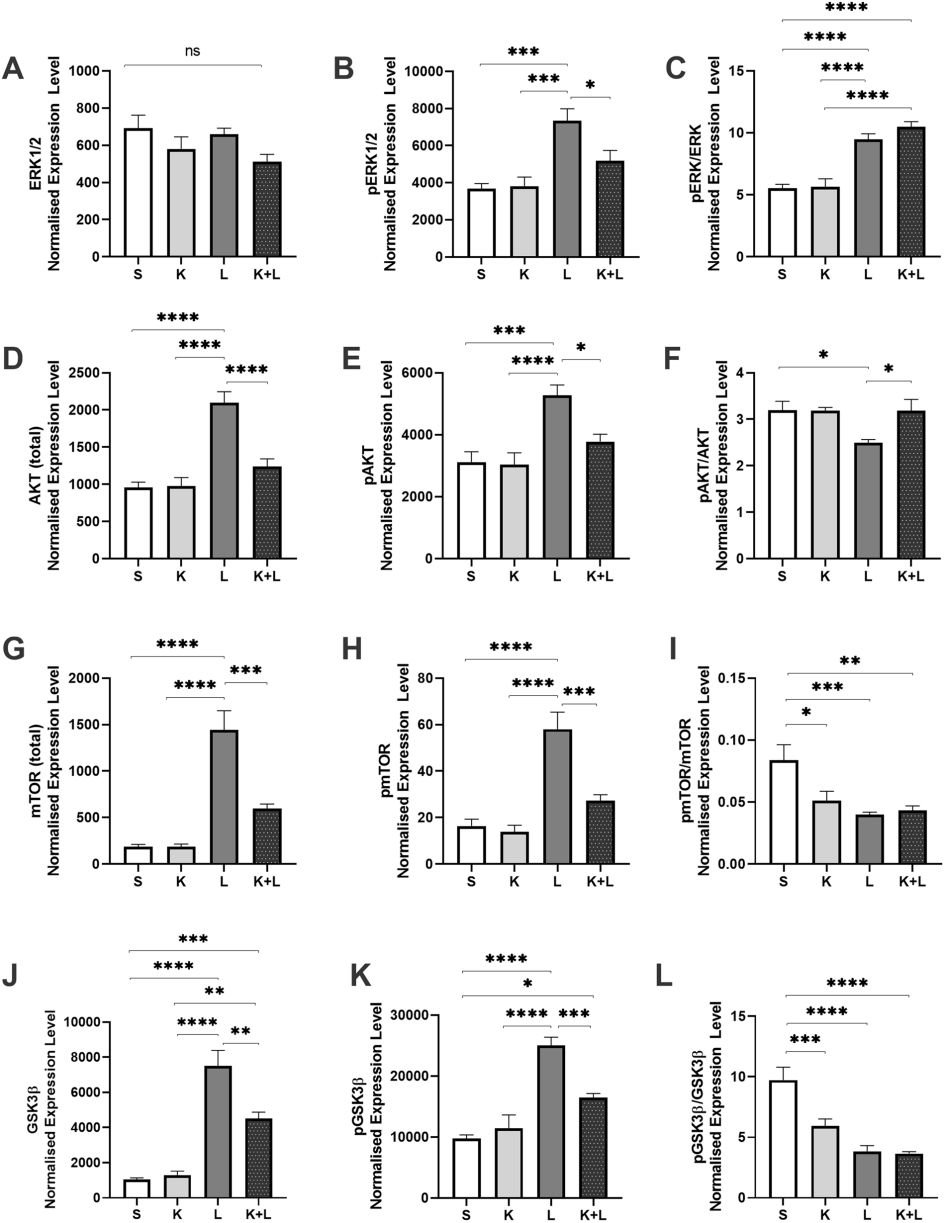
Prelimbic cortex protein expression in response to treatment. The effect of ketamine and lithium treatment on protein signaling profiles in the infralimbic cortex were examined via western blotting. Proteins of interest include total ERK1/2 (**A**), *p*ERK1/2 (**B**), *p*ERK1/2/ERK1/2 (**C**), total Akt (**D**), *p*Akt (**E**), *p*Akt/Akt (**F**), mTOR (**G**), *p* mTOR (**H**), and *p* mTOR/mTOR (**I**), GSK3ß (**J**), *p*GSK3ß (**K**), and *p*GSK3ß/GSK3ß (**L**). ERK1/2 extracellular signal-regulated kinases 1/2, *p* phosphorylated, Akt protein kinase B, mTOR mammalian target of rapamycin, GSK3ß glycogen synthase kinase-3 ß. Treatment groups: S: saline, K: ketamine, L: lithium, K + L: ketamine + lithium. *p < 0.05; **p < 0.01; ***p < 0.001; ****p < 0.0001.

A significant main effect of ketamine treatment (*p* = 0.0156; *F* = 6.366) was observed for ERK1/2 signaling in the PL PFC, however there were no significant group differences following post hoc multiple comparisons (Fig. 4A). A significant main effect for lithium treatment (*p* < 0.0001; *F* = 22.56) and treatment interaction was observed for *p*ERK1/2 (*p* = 0.0359; *F* = 4.7), with lithium treated animals showing significantly greater levels relative to control (*p* = 0.0001), ketamine (*p* = 0.0001), and ketamine + lithium (*p* = 0.0293) treated animals (Fig. 4B). A robust and significant main effect for lithium treatment (*p* < 0.0001; *F* = 78.38) was also seen for the ratio of *p*ERK1/2 to total ERK1/2. Post hoc comparisons revealed that animals treated with lithium and animals receiving the combination of lithium with ketamine demonstrated significant greater levels of *p*ERK/ERK relative to control or ketamine treated animals, with *p* < 0.0001 for each of these comparisons (Fig. 4C).

##### Total and phosphorylated Akt

A significant main effect was apparent for the effects of ketamine (*p* = 0.0007; *F* = 13.29) and lithium (*p* < 0.0001; *F* = 36.95) on Akt. A significant treatment interaction was also present (*p* = 0.0004; *F* = 14.51). Post hoc analyses revealed levels of Akt were significantly higher in lithium treated animals relative to control animals (*p* < 0.0001), animals treated with ketamine (p < 0.0001) and animals treated with the combination of ketamine and lithium (*p* < 0.0001) (Fig. 4D). Similarly, a significant main treatment effect was observed for the effects of ketamine (*p* = 0.0208; F = 5.78) and lithium (*p* < 0.0001; *F* = 19.60) on *p*Akt expression, together with a significant treatment interaction effect (*p* = 0.0372; *F* = 4.638). Post hoc tests demonstrated the animals receiving lithium had significantly higher levels of *p*Akt relative to control saline (*p* = 0.0003), ketamine (*p* < 0.0001), and ketamine with lithium (*p* = 0.0114) (Fig. 4E). A borderline significant main effect was observed for effects of lithium (*p* = 0.0544; *F* = 3.947) on *p*Akt/Akt, as well as for treatment interaction (*p* = 0.00653; *F* = 3.977). Post hoc multiple comparisons revealed that lithium treated animals had significantly less *p*Akt/Akt compared to vehicle-treated control animals (*p* = 0.0391) and animals receiving ketamine and lithium co-treatment (*p* = 0.0325). Expression levels of *p*Akt/Akt were also lower in lithium treated animals relative to animals receiving ketamine, though significance was borderline (*p* = 0.052) (Fig. 4F).

##### Total and phosphorylated mTOR

Significant main treatment effects of ketamine (*p* = 0.0023; *F* = 10.77) and lithium (*p* < 0.0001; *F* = 41.68) on mTOR were observed. A significant treatment interaction was also present was observed for mTOR (*p* = 0.0024; *F* = 10.68). Multiple comparisons revealed animals receiving lithium had significantly higher levels of mTOR relative to control saline (*p* < 0.0001), ketamine (*p* < 0.0001), and ketamine with lithium (*p* = 0.0001) (Fig. 4G). Similarly for treatment effects on *p*mTOR, a significant main effect was evident for ketamine (*p* < 0.0001; *F* = 33.31) and lithium (p = 0.0012; F = 12.20), together with a significant treatment interaction (*p* = 0.005; *F* = 8.865). Posthoc analyses showed that PL PFC levels of *p*mTOR were significantly higher in lithium treated animals relative to animals receiving saline vehicle (*p* < 0.0001), ketamine (*p* < 0.0001) or ketamine and lithium co-treatment (p = 0.0002). A significant main effect for ketamine (*p* = 0.0487; *F* = 4.146) and lithium (*p* = 0.0008; *F* = 13.24) on *p*mTOR/mTOR expression was also evident, together with a significant interaction effect (*p* = 0.0159; *F* = 6.673). Multiple comparisons revealed that relative to control animals the ratio of *p*mTOR/mTOR was significantly decreased in animals treated with ketamine (*p* = 0.0138), lithium (*p* = 0.0007), or the combination of ketamine with lithium (*p* = 0.0025) (Fig. 4I).

##### Total and phosphorylated GSK3ß

Significant main effects for both ketamine (*p* < 0.0001; F72.60) and lithium (*p* = 0.0199; *F* = 5.909) were found for GSK3ß expression, together with an interaction effect (*p* = 0.0069; *F* = 8.147). Post hoc comparisons revealed that control animals had significantly lower levels of GSK3ß compared to animals treated with lithium (*p* < 0.0001) and the lithium + ketamine combination (*p* = 0.0006). Animals treated with ketamine had similarly reduced levels of GSK3ß relative to animals receiving lithium (*p* < 0.0001) or ketamine + lithium (*p* = 0.0015). Levels of PL GSK3ß were also high in animals treated with ketamine relative to those receiving the combination treatment (*p* = 0.0014) (Fig. 4J). Significant treatment (ketamine: *p* < 0.0001; *F* = 48.33; Lithium: *p* = 0.0242; *F* = 5.48) and interaction (*p* = 0.001; *F* = 12.52) main effects were also present for *p*GSK3ß. Relative to controls, PL levels of *p*GSK3ß were increased in animals receiving lithium (*p* < 0.0001) or lithium with ketamine (*p* = 0.0005) (Fig. 4K). Similarly, main treatment (ketamine: *p* < 0.0001; *F* = 43.03; Lithium: *p* = 0.0027; *F* = 10.23) and interaction (*p* = 0.006; *F* = 8.4) effects were seen for PL *p*GSK3ß/GSK3ß, with significantly lower levels of expression in ketamine (0.0007), lithium (*p* < 0.0001) and ketamine + lithium (*p* < 0.0001) treated animals relative to controls. Trends towards significantly higher levels of PL *p*GSK3ß/GSK3ß were also seen in animals receiving lithium (*p* = 0.0814) alone or in combination with ketamine (0.0568), relative to animals receiving only ketamine (Fig. 4L).

### Correlations between behavioral and molecular markers

#### Antidepressant-like response and peripheral blood markers

**Fig. 5:**
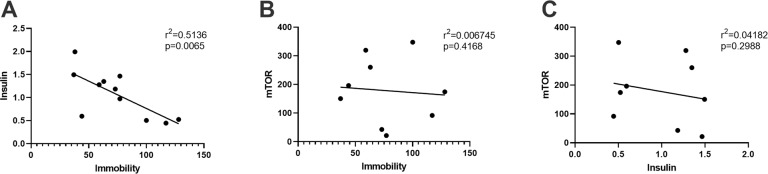
Correlation between antidepressant-like response and peripheral blood markers. A significant negative correlation was observed between levels of insulin in peripheral blood (plasma) and time spent immobile in the forced swim test in animals receiving both ketamine and lithium (**A**). No linear relationship was observed for peripheral levels of mTOR and immobility time (**B**), nor for peripheral mTOR and insulin (**C**). mTOR mammalian target of rapamycin.

In animals receiving both ketamine and lithium, a significant negative correlation was observed between time spent immobile in the forced swim test and peripheral insulin levels acquired 1 h after the test (*r*^2^ = 0.5136; *p* = 0.0065). No significant linear relationship was observed for peripheral levels of mTOR and immobility time, nor for peripheral levels of mTOR and insulin (Fig. 5). No relationship between was observed between insulin, mTOR and immobility time for any of the other treatment groups.

#### Antidepressant-like response and pERK/ERK expression

**Fig. 6:**
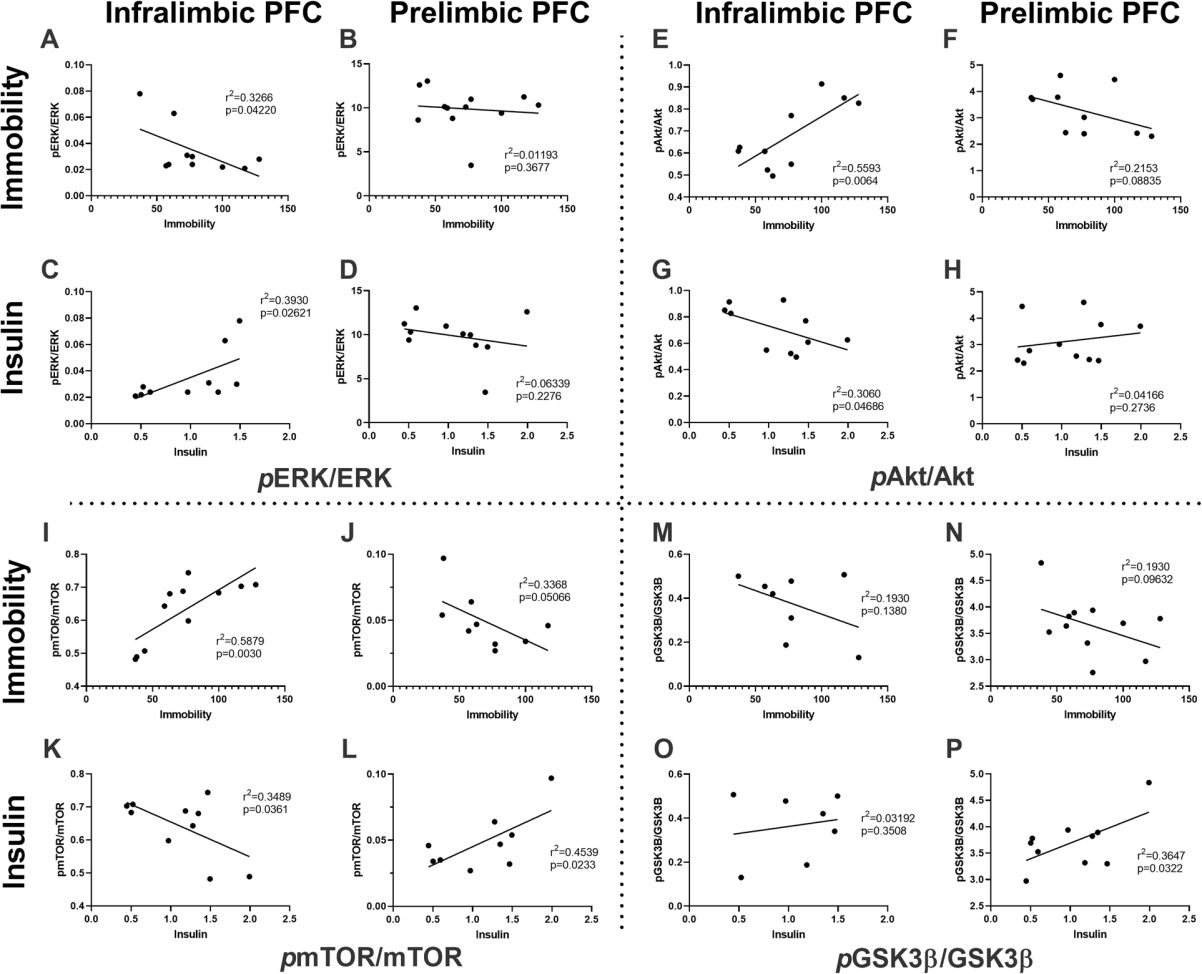
Correlation between antidepressant-like response or peripheral insulin levels and prefrontal protein expression. The relationship between phosphorylated (*p*) protein expression as a ratio of total protein expression in the infralimbic and prelimbic prefrontal cortex (columns) and time spent immobile in the forced swim test or peripheral blood insulin levels (rows) for extracellular signal-regulated kinases 1/2 (ERK1/2; **A**–**D**); protein kinase B(Akt; **E**–**H**); mammalian target of rapamycin (mTOR; **I**–**L**); and, glycogen synthase kinase-3ß (GSK3ß; **M**–**P**).

In animals receiving both ketamine and lithium, a significant negative correlation was observed between time spent immobile in the forced swim test and *p*ERK/ERK expression levels in the IL PFC (*r*^2^ = 0.3266; *p* = 0.0422; Fig. 6A). No significant relationship was observed between immobility time and *p*ERK/ERK expression levels in the PL (Fig. 6B). Insulin levels were positively correlated with *p*ERK/ERK expression in the IL (*r*^2^ = 0.3930; *p* = 0.02621; Fig. 6C), but not PL (Fig. 6D).

#### Antidepressant-like response and pAkt/Akt expression

A significant positive correlation was observed between time spent immobile and *p*Akt/Akt expression levels in the IL PFC (*r*^2^ = 0.5593; *p* = 0.0064; Fig. 6E) of animals receiving both ketamine and lithium. Contrasting this, there was a nonsignificant negative linear relationship between immobility time and *p*Akt/Akt (*r*^2^ = 0.2153; *p* = 0.08835; Fig. 6F) in the PL PFC of these animals. Insulin levels were negatively correlated with *p*Akt/Ak expression in the IL (*r*^2^ = 0.3060; *p* = 0.04686; Fig. 6G), but not PL (Fig. 6H).

## Discussion

Lithium augmentation produced an antidepressant-like response in ketamine non-responsive animals, as demonstrated by its acute enhancement of active coping behavior under stress. We have previously shown that this ACTH-pretreated animal model of TRD exhibits ketamine responsive and non-responsive phenotypes [24], similar to clinical cases [10]. In the current study, animals predominantly demonstrated the nonresponsive phenotype, while those receiving lithium augmentation of ketamine displayed robust antidepressant responses. Alone, however, lithium was ineffective in eliciting an antidepressant response which aligns with previous observations in this model demonstrating lithium is not sufficient to enable active stress coping in the forced swim test, yet can shift this behavioral response when administered adjunctive to an antidepressant [15, 25]. Of particular interest was the observation that animals co-treated with ketamine and lithium expressed elevated biochemical markers, including higher plasma levels of insulin and mTOR, together with the significant upregulation of insulin signaling in the IL PFC, relative to other nonresponsive groups. This increase in insulin signaling proteins contrasted the predominant downregulation observed in the PL PFC. Moreover a contrasting effect on pERK/ERK was observed in each region, with decreases and increases observed in the IL and PL, respectively, of those animals receiving either lithium or the ketamine+lithium combination. Moreover, this pattern of expression directly correlated with plasma insulin and behavioral antidepressant-like response in antidepressant-resistant animals receiving lithium augmentation of ketamine. Our previous work suggests that lithium augmentation may facilitate neurotrophic cellular responses to insulin to improve active coping behavior in the forced swim antidepressant response behavioral assay [15], as well as contribute to improved therapeutic response to lithium in the clinic [34].

Insulin signaling plays and important role in promoting neuroplasticity, neurogenesis and neuroprotection [15, 16, 35] while also enabling the efficient utilization of metabolic machinery for bioenergetic output [3, 18, 36]. We therefore posited that mitochondrial responses to neurotrophic and inflammatory mediators would be affected by each treatment protocol and assessed effects on OCR and ECAR, measures of the overall oxidative and nonoxidative capacity, respectively. However, compared to controls, animals receiving the combination of ketamine and lithium showed no differences in either oxidative metabolism (OCR) or glycolytic activity (ECAR) in response to BDNF or TNFα stimulation. In contrast, animals administered lithium showed elevated ECAR responses to BDNF relative to control animals, suggesting that lithium may be engaging glycolytic metabolism to enhance bioenergetic output in response to BDNF. These same lithium treated animals, also exhibited a significant increase in TNF-α mediated OCR relative to animals receiving both ketamine and lithium. While these bioenergetic effects were not associated with any antidepressant-like behavioral effects in the current protocol, they suggest that lithium functionally regulates metabolic responses to neurotrophic and pro-inflammatory stimuli. This may have important implications for coordinating cellular responses to these factors, via gating of metabolic pathways. It is important to also note that these assays were performed in PFC tissue anterior to the IL and PL, thereby lacking the same regional specificity used for the molecular signaling assays. However, the functional nature of these assays, still serves to provide useful insight into how BDNF and TNFα impact metabolic phenotype within the PFC and suggests an important role of lithium in moderating these effects.

Lithium administration elevates mitochondrial biogenesis in the central nervous system and promotes the expression of mitochondrial and endoplasmic reticulum proteins essential for regulating apoptosis [16, 37, 38]. These actions serve to mitigate the negative effects of excessive reactive oxygen species (ROS) on mitochondria, enhancing cellular resilience to stress [16]. When coupled with increased glycolytic sensitivity to BDNF, this increased metabolic capacity and cellular resilience may increase neuronal capacity for plasticity following ketamine. While promotion of synaptic plasticity is tied to the antidepressant effects of ketamine [4, 5], the lack of behavioral response to lithium and the lack of metabolic response to ketamine in the present study may indicate that ketamine’s promotion of synaptic plasticity arrives via cellular mechanisms that do not directly engage mitochondria and/or glycolytic rate—perhaps instead involving the promotion of peripheral insulin and consequent moderation of insulin signaling (upregulation of mTOR and Akt phosphorylation) in the IL and PL, a regional mechanism of promoting antidepressant-like responsivity in the ACTH-pretreated model of TRD [15].

Animals displaying a nonresponsive antidepressant phenotype to ketamine alone, similarly failed to significantly differentiate from untreated control animals on biological measures. Importantly, time spent immobile, indicating lack of antidepressant efficacy, was negatively correlated with IL insulin signaling across treatments. This suggests that the augmentative effects of lithium as an adjunctive therapy for ketamine may help promote insulin signaling associated with antidepressant response to enable antidepressant responsivity in otherwise nonresponsive individuals. A similar correlation was observed in our prior work demonstrating lithium augmentation of imipramine promoted antidepressant response the ACTH-pretreated rodent model of TRD together with upregulation of central and peripheral insulin signaling [15]. In the current study, the upregulation of insulin signaling with combinatorial treatment may functionally promote cell growth, synaptic protein synthesis and neuroplasticity/protection [15, 35, 39], while elevated insulin levels may serve as a bioenergetic resource [19]. Thus, while ketamine had a reduced response rate as a solo treatment, it may be that the addition of lithium served to improve the overall response rate through coordination of a cascade of insulin-stimulated neurotrophic and intracellular signals to ultimately enhance antidepressant responsivity.

### Clinical implications

Combinatorial ketamine and lithium co-treatment yielded a consistent antidepressant-responsive phenotype within a pathophysiological context modeling TRD. In particular, these data suggest that lithium augmentation, via the upregulation of insulin and insulin signaling, enabled antidepressant response in otherwise ketamine nonresponsive individuals. Adding lithium augmentation to individuals nonresponsive to ketamine may similarly improve therapeutic outcomes in the clinic. While lithium augmentation of ketamine has recently been trialed in the clinic, this was done selectively in patients demonstrating at least an initial partial response to a single intravenous infusion of ketamine (0.5 mg/kg) [40]. These ketamine responsive individuals were then randomized under double-blind conditions to receive either lithium or placebo together with an additional three infusions of ketamine. Under these conditions, no difference in depression severity or duration of antidepressant response was observed between treatment groups [40]. While the observed failure of lithium to improve clinical outcomes to ketamine is disappointing, the results of the current study suggest instead that enhanced efficacy may have instead been observed had individuals initially nonresponsive to the first ketamine infusion been recruited to receive lithium augmentation. That is, lithium augmentation of ketamine may only be necessary and beneficial when there is an inherent deficit in critical moderators of response, such as insulin signaling, which can be facilitated with supplemental target engagement. In line with this, in a naturalistic study, drug levels of lithium did not correlate with ketamine’s antidepressant efficacy in treatment-resistant bipolar depression [37]. This further suggests that the addition and amount of lithium adjunctive to ketamine is not the most clinically relevant factor. Rather, it may be more critical to focus on how the drug moderates response by promoting molecular signaling cascades and bioenergetic pathways essential for enabling antidepressant responsivity. Clinical studies using this precision medicine approach are needed to confirm the potential for lithium augmentation to promote a therapeutic response in clinical samples otherwise nonresponsive to ketamine.

## Conclusions

Lithium, a commonly prescribed mood stabilizer, may be useful for clinicians as an augmentative tool to improve antidepressant outcomes to ketamine. Here, we have presented evidence in support of the potential for lithium to enable antidepressant response to ketamine in a rodent model of TRD, using a cohort primarily nonresponsive to ketamine alone. Moreover, we present important new data demonstrating that, in combination, these treatments upregulated insulin signaling in the IL, indicating that this may be an important molecular therapeutic target. These results hold promise for clinical application and highlight the need to move forward with individualized target engagement studies that will enable progress towards precision medicine for psychiatry. By enabling individualized targeting of key rate limiting molecular mechanism, this approach has the potential to reshape our understanding of TRD, suggesting that failed drug efficacy may have more to do with misalignment of drug target with physiology. The capacity to enable response to the rapid acting antidepressant actions of ketamine in otherwise ‘treatment resistant’ individuals, remains an important goal for future research.
